# T-regulatory cells for the treatment of autoimmune diseases

**DOI:** 10.3389/fimmu.2025.1511671

**Published:** 2025-02-04

**Authors:** Marina S. Fisher, Sergey V. Sennikov

**Affiliations:** ^1^ Laboratory of Molecular Immunology, Federal State Budgetary Scientific Institution Research Institute of Fundamental and Clinical Immunology, Novosibirsk, Russia; ^2^ Laboratory of Immune Engineering, Federal State Autonomous Educational Institution of Higher Education I.M. Sechenov First Moscow State Medical University under the Ministry of Health of the Russian Federation (Sechenov University), Moscow, Russia

**Keywords:** T-regulatory cells, autoimmune diseases, immunological tolerance, polyclonal Tregs, CAR-Treg, TCR-Treg, antigen-specific therapy

## Abstract

Autoimmune diseases result from imbalances in the immune system and disturbances in the mechanisms of immune tolerance. T-regulatory cells (Treg) are key factors in the formation of immune tolerance. Tregs modulate immune responses and repair processes, controlling the innate and adaptive immune system. The use of Tregs in the treatment of autoimmune diseases began with the manipulation of endogenous Tregs using immunomodulatory drugs. Then, a method of adoptive transfer of Tregs grown *in vitro* was developed. Adoptive transfer of Tregs includes polyclonal Tregs with non-specific effects and antigen-specific Tregs in the form of CAR-Treg and TCR-Treg. This review discusses non-specific and antigen-specific approaches to the use of Tregs, their advantages, disadvantages, gaps in development, and future prospects.

## Introduction

Autoimmune diseases (AIDs) arise as a result of an imbalance in the immune system and impairments in the mechanisms of immune tolerance. To a large extent, the state of the immune system depends on T-regulatory cells (Tregs), which are key factors of immune tolerance. In addition to immunoregulatory properties, Tregs participate in the regenerative processes of skeletal and cardiac muscles, skin, lungs, bones, and the central nervous system (1).

Tregs are characterized by the expression of markers CD4 and CD25, as well as by inhibiting the activation and proliferation of helper CD4^+^ T cells, cytotoxic CD8^+^ T cells, and prevention of B cell activation. All Tregs can be divided into two groups including natural Tregs (nTregs) and induced Tregs (iTregs) (2). The cell-specific marker of nTreg and some iTreg subpopulations is FOXP3, which is necessary for their maturation and function. Further, iTregs are divided into four more subpopulations, namely IL-10-secreting CD4^+^ Treg1 cells (Tr1 cells), TGF-β-secreting Tregs (Th3), CD8^+^ Tregs, and CD4^+^CD25^+^FoxP3^–^IL-35-dependent cells (iTr35) (3). Meanwhile, nTregs originate from CD4^+^ thymocytes and leave the thymus as CD4^+^CD25^+^FoxP3^+^ cells.

Tregs can modulate immune responses and reparative processes through control of both the innate and adaptive immune systems.

**Figure 1:**
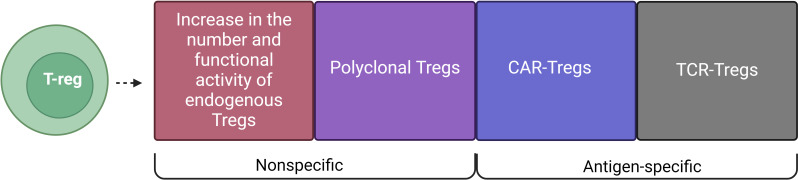
Types of Treg cell therapy for AIDs. T-regulatory cell-based therapies include: Increase in the number and functional activity of endogenous Tregs and Polyclonal Tregs, which are non-specific methods, and CAR-Tregs and TCR-Tregs, which are antigen-specific methods.

The involvement of Tregs in the treatment of AIDs began with the attempt of the impact on endogenous Tregs (**Figure 1**). In fact, the introduction of immunomodulatory agents was used to increase the number and/or functional activity of endogenous Tregs *in vivo*. Later, another approach emerged, which involved the adoptive transfer of *in vitro* expanded Tregs. Adoptive transfer of Tregs consists of several variations: polyclonal Tregs with a non-specific impact on the immune system and antigen-specific Tregs in the form of CAR-Tregs and TCR-Tregs. This review highlights non-specific and antigen-specific approaches to the use of Tregs.

## The effect of Tregs on immune system cells to implement immunosuppressive function

**Figure 2:**
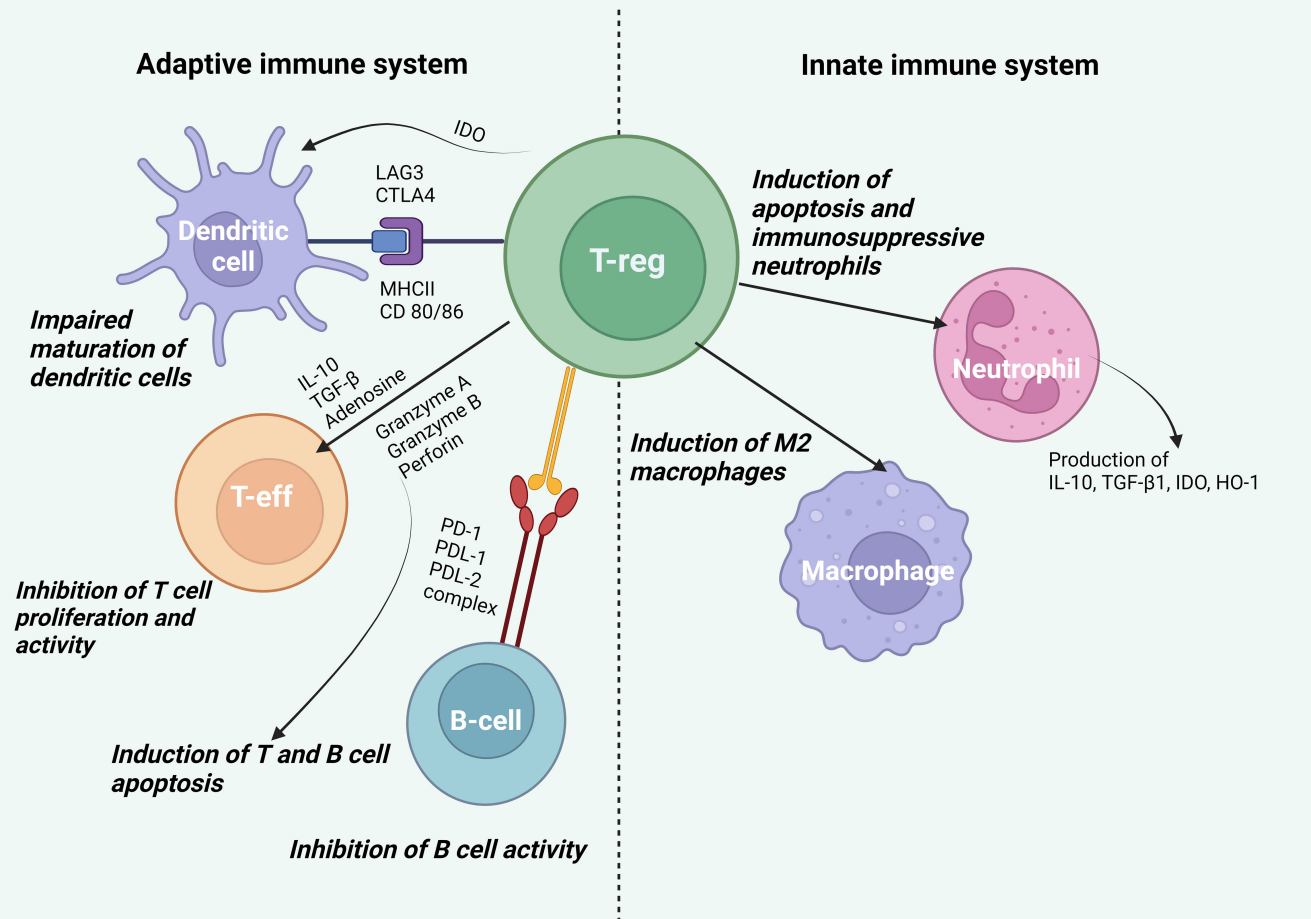
Effect of Tregs on cells of the innate and adaptive immune systems. *Effect on cells of the innate immune system*. Tregs induce neutrophil apoptosis, promote the formation of secondary immunosuppressive neutrophils generating IL-10, TGF-β1, IDO, and HO-1, and stimulate neutrophil phagocytosis by macrophages and polarization of macrophages into the anti-inflammatory phenotype M2. *Effect on cells of the adaptive immune system*. Tregs bind LAG3 to MHCII and CTLA-4 to CD80/86 on DCs, which disrupts the maturation and antigen presentation processes in DCs promoting the formation of DCs with a tolerogenic phenotype. By producing IDO, Tregs disrupt the binding of CD80/86 on DCs to CD28 on T effector cells, inhibiting their activation. IL-10 directly inhibits T cell expansion, suppressing antigen presentation and DC activity, while TGF-β blocks T lymphocyte proliferation and Th1 and Th2 cell differentiation. Tregs suppress metabolic processes by producing adenosine leading to suppression of T cell activity. Tregs regulate the activity of autoimmune B lymphocytes through the binding of PD-1 on autoimmune B cells to PDL-1 and PDL-2 on Tregs. Additionally, Tregs can use granzyme A and B as well as perforin to indirectly suppress the activity of effector B lymphocytes and T cells.

First of all, it should be noted that Tregs can affect cells of the innate immune system including macrophages and neutrophils, which are involved in inflammatory and regenerative processes (**Figure 2**). The study by Lewkowicz et al. (4) showed that Tregs induced neutrophil apoptosis and blocked the production of IL-6 by neutrophils, as well as caused the formation of secondary immunosuppressive neutrophils, generating IL-10, TGF-β1, IDO, and HO-1. The authors of the study (5) also demonstrated neutrophil apoptosis induced by Tregs. Besides, Tregs could stimulate neutrophil phagocytosis by macrophages and promote macrophage polarization to the M2 phenotype by anti-inflammatory cytokines (e.g., IL-4, IL-10, IL-13) (1, 6).

Once activated, Tregs can impact dendritic cells (DCs), T effector cells, and B cells (**Figure 2**). Tregs bind LAG3 to MHCII and CTLA-4 to CD80/86 on DCs, thereby disrupting the maturation and antigen presentation processes on DCs and promoting the formation of DCs with a tolerogenic phenotype. By producing IDO, Tregs also disrupt the binding of CD80/86 on DCs to CD28 on T effector cells and block their activation (7). IL-10, produced by Tregs, directly inhibits T cell expansion and suppresses antigen presentation and DC activity (8). TGF-β, which is also produced by Tregs, can block T cell proliferation by inhibition of the IL-2 expression via the Smad3 signaling pathway, as well as by inhibition of cyclins including cyclin D2 and cyclin E, cyclin-dependent kinase (CDK-4) and c-myc. TGF-β can inhibit Th1 and Th2 cell differentiation by blocking the T-bet/STAT4 and GATA-3/NFAT signaling pathways (9). Tregs are able to induce apoptosis of effector cells through the release of granzyme A, granzyme B, and perforin. Activated Tregs can suppress metabolic processes by generating adenosine from ATP, which is further metabolized through the CD39/CD73 pathway. This process entails T cell suppression by inducing negative signaling towards antigen-presenting cells (APCs) and effector T cells (10). Due to the high surface expression of CD25 and the ability to scavenge IL-2, Tregs can reduce proinflammatory cytokine signaling (11).

Recent research has revealed that Tregs play a crucial role in suppressing autoimmune B-lymphocyte activity. To achieve this effect, Tregs require two key conditions: high expression of PD-1 on autoimmune B cells and simultaneous activity of two molecules, PDL-1 and PDL-2, which bind to PD-1 (12). This enables Tregs to control the activity of autoimmune B lymphocytes. Additionally, Tregs can use granzyme B and perforin to indirectly suppress the activity of effector B lymphocytes. These molecules are able to reduce the production of autoantibodies ultimately leading to a decrease in the activity of autoimmune B lymphocytes (12).

The features of antigen-specific Tregs are of particular interest. Antigen-specific Tregs are activated upon recognition of specific antigens presented by APCs. This targeted activation allows them to suppress effector T cells, which respond to the same antigens, and effectively prevent inappropriate immune responses against self-tissues. Antigen-specific Tregs are more effective in controlling autoimmune reactions since they can migrate to tissues containing their cognate antigens (13, 14). This localized action reduces the risk of non-specific immunosuppression, making them a promising therapeutic option.

The antigen-specific mechanism of action of Tregs is critical to their role in immune regulation. Tregs can effectively suppress autoreactive T cells and maintain immune homeostasis. This specificity not only enhances their therapeutic potential in AIDs but also determines the importance of comprehension of their biology for the development of Tregs-based targeted therapy.

Based on the above, it becomes clear that Tregs are indeed a promising platform for the development of AID therapy.

## Types of therapy based on the use of Tregs for AIDs

As mentioned earlier, there are two main methods of impact on Tregs: administration of immunomodulatory agents, which increase the number and/or efficacy of Tregs *in vivo*, and adoptive transfer of *in vitro* expanded Tregs (13) (**Table 1**).

**Table 1:** Tregs-based therapies for autoimmune diseases: targets, specificity, advantages and disadvantages.

Approaches	Stimulation of Endogenous Tregs	Polyclonal Tregs	CAR-Tregs	TCR-Tregs
Specificity	Non-specific	Non-specific	Specific	Specific
Induction method	Endogenous Tregs	Expansion of polyclonal Tregs	Recognition of target antigen on the surface of target cells via the CAR structure; synthetic receptor signal	Intracellular interaction of peptide–MHC with TCR; endogenous TCR signaling
Advantages	Effect only on endogenous cells, without adoptive transfer	Impact on the immune system through a wide pool of expanded Tregs, proven safety of using	MHC-independent antigen recognition that provides broader applicability	More stable, with low levels of inflammatory cytokines; greater efficacy compared to polyclonal Tregs; the only interaction of the peptide-MHC with TCR is required for Treg to be activated; are specific and personalized that eliminates the risk of developing non-specific immunosuppressive reactions; fewer cells are needed to implement the effect compared to polyclonal Tregs
Disadvantages	General immunosuppression; activation of T-effector cells;	General immunosuppression; lack of efficacy in clinical trials; difficulty in obtaining sufficient cell numbers for therapy; need for large numbers of cells to achieve effect	Higher levels of inflammatory cytokines; requirement for expression of at least 100 target autoantigens on the target cell surface for Treg activation; potential for non-specific immunosuppressive response due to hyperactivation of Treg in non-target tissues	MHC-restricted antigen recognition, leading to more personalized therapy; selection of sources for TCR isolation is required; determination of TCR affinity rate for therapy efficacy; possibility of miscoupling of endogenous and transduced TCR chains

Currently, several methods are used to increase endogenous Tregs. These include low doses of IL-2, mutant IL-2, IL-2/anti-IL-2 complexes, rapamycin as the mTOR inhibitor, and gut microbiome transplantation. Several studies have shown the efficacy of low doses of IL-2 (15–17). The advantage of IL-2 is associated with its common availability, for example, in the form of Proleukin (Aldesleukin). However, it is important to remember that, depending on the dose, IL-2 therapy can lead to an increase in the number of not only Tregs, but also T-effector cells (18). Rapamycin has been shown to inhibit mTOR (19, 20), a molecule involved in triggering the activation signal from IL-2R and CD28 via phosphatidylinositol 3-kinase (PI3K), thereby blocking the activation signal in T cells and their proliferation (21, 22). Thus, rapamycin selectively increased the number of Tregs while maintaining their suppressor phenotype (19). Gut microbiome transplantation is considered a method for stimulating Tregs. Studies have shown that butyrate, produced by certain types of bacteria in the gut, was able to regulate the expression of anti-inflammatory genes in DCs and increased the stability of expression of the transcription factor FOXP3 in Tregs (23, 24).

Adoptive transfer is based on the isolation of Tregs from peripheral blood and their expansion *in vitro*. Tregs can be isolated using CliniMACS reagents, which use double negative selection (monoclonal antibodies against CD8 and CD19) followed by positive selection (monoclonal antibodies against CD25). The study by Mauro Di Ianni et al. (25) emphasized that this method enriches CD4+CD25+FoxP3+ cells with immunosuppressive capabilities, which can serve as a source of natural Tregs lacking CD8+ and CD4+/CD25- clones. It is also possible to sort Treg cells using the FACSAria device, after staining the cells with a cocktail of monoclonal antibodies, as in the study Trzonkowski et al. (26). Polyclonal expansion of Tregs is mostly achieved by addition of beads coated with monoclonal antibodies against CD3/CD28 in the presence of IL-2 (27), with or without rapamycin. Furthermore, the co-use of TGF-β and all-trans retinoic acid (ATRA) (28–30) or vitamin D and TGF-β has been discribed. Adoptive transfer of Tregs involves several approaches including polyclonal Tregs and antigen-specific Tregs.

### Polyclonal regulatory T cells

Currently, therapy with Tregs is well characterized. The first data on the use of polyclonal T-regs were promising and focused on graft-versus-host disease (GVHD) in 2009 (26) and Crohn’s disease in 2012 (31). The therapy was safe and resulted in a reduction in the severity of symptoms in Crohn’s disease, while in GVHD it decreased the need for immunosuppressive therapy. Subsequently, many scientists conducted research in this area. In 2015, Theil et al. (32) developed a GMP protocol for isolating and expanding stem cell donor-derived Tregs. They also demonstrated the feasibility of clinical use of these Tregs in five patients with chronic GVHD. In 2022, Landwehr-Kenzel et al. conducted a clinical trial using polyclonally expanded Tregs in three children with severe GVHD that was refractory to treatment (33). All children showed clinical improvement and decreased GVHD activity. Clonal expansion of Tregs was confirmed by next-generation sequencing.

Afterwards, polyclonal Tregs were used for the treatment of AIDs. For example, in patients with type 1 diabetes mellitus (T1DM) treated with T-regs, insulin requirements and C-peptide levels remained stable although no reduction in disease progression was observed (34–36). Bender et al. also showed insufficient efficacy of polyclonal Tregs therapy in patients with T1DM: single doses did not prevent the decline in residual β-cell function over 1 year compared with placebo (37). In systemic lupus erythematosus (SLE), the use of polyclonal T-regs resulted in temporary stabilization of the disease but injected T-regs were detected in skin biopsies of a patient with active skin disease (38). Thus, efficacy was partial and insufficient to significantly improve the course of the diseases.

Over time, a problem, which might have caused the lack of efficacy of this type of therapy, was revealed, and it was stipulated by the non-specificity of the polyclonal Treg effects. A number of preclinical studies demonstrating the relationship between the antigen specificity of Tregs and their therapeutic efficacy have shown that antigen-specific Tregs were more effective than polyclonal Tregs (39–43).

Another challenge in the therapy with polyclonal Tregs is obtaining a sufficient number of cells with a stable phenotype. To eliminate these problems, several strategies are being developed; these include stabilization of the FOXP3 gene expression with Cas9 and Helios proteins, epigenetic editing of FOXP3, protection of FOXP3 from polyubiquitination, and enhancing the suppressive ability of Tregs by RNA interference targeting protein kinase PKCθ (44). In addition, the co-use of polyclonal Tregs and standard immunosuppressive therapy reduced the efficacy of this type of cell therapy (45). To make Tregs resistant to immunosuppressant drugs, Amini and other researchers developed a protocol based on the CRISPR-Cas9 system that effectively targets the FKBP12 gene, an adaptor protein that is essential for the immunosuppressive function of tacrolimus (46).

In the review by Amini et al., the term “Super Tregs” is introduced, referring to genetically modified Tregs with enhanced capabilities to more effectively suppress immune responses. Various genetic engineering tools exist that allow precise manipulation of Tregs. These include gene editing using programmable nuclease systems (CRISPR/Cas9, TALENs, and ZFNs), delivery of gene editing components, and various genetic engineering strategies to enhance Treg stability and function (47).

### Antigen-specific regulatory T cells

Antigen-specific Tregs can be produced *in vitro* by genetically inserting synthetic receptors, including engineered T cell receptors (TCRs) and chimeric antigen receptors (CARs). CAR and TCR therapies have been rapidly developing in the last decade. Until recently, these developments were used to treat cancer but now these technologies have become of interest for the treatment of AIDs.

#### CAR technology

It should be noted that the use of CAR technology in AIDs began with CAR-T cells. Research and clinical trials of CAR-T therapy for the treatment of AIDs have shown its potential efficacy (48, 49). In particular, a recent study involved patients with SLE (50). Autologous T cells from SLE patients were transduced with a lentiviral CAR vector against CD19, expanded, and reinfused. As a result, remission was achieved in 3 months, and drug-free remission lasted more than 8 months. Another study followed fifteen patients with three different AIDs including SLE, idiopathic inflammatory myositis, and systemic sclerosis for two years; they were treated with a single infusion of T cells with CD19-chimeric antigen receptor (51). All patients experienced significant clinical responses but some of them had side effects such as cytokine storm, neurotoxicity, and pneumonia.

However, despite its potential efficacy, CAR-T cell therapy has a number of challenges and risks. Uncontrolled activation and proliferation of CAR-T cells can lead to undesirable consequences, such as cytokine storm and thrombocytopenia (52). In addition, there is a risk of toxic effects on organs and tissues due to the uncontrolled destruction of healthy cells as well as a risk of autoimmune reactions during the migration and proliferation of CAR-T cells that leads to the development of new AIDs (52).

To sort out the problems associated with the CAR-T, one should pay attention to the use of CRISPR–Cas9 technology (53). For example, modified CRISPR–Cas9 CAR-T with dual targeting to CD19 and CD22 antigens was successfully used for the treatment of 11 patients with B-cell acute lymphoblastic leukemia (54). No cases of hepatotoxicity or infectious complications were observed during the treatment. This approach demonstrated a safe profile and high antileukemic activity.

Another effective approach to solve these problems is associated with CAR-Tregs. The source for producing CAR-Tregs can be CD4^+^ T cells or Tregs (55). Obtaining CAR-Tregs by means of polyclonal Tregs has limitations, such as low levels of Tregs in the peripheral blood and an unstable phenotype (55). It is possible that the use of CD4^+^ T cells and viral transduction of FOXP3 is the most promising as it results in the production of cells with a more stable phenotype and bypasses the problem of low Tregs in the peripheral blood (55–57).

It is suggested that through clonal deletion and induction of anergy, CAR-Tregs can reveal and regulate the activity of autoimmune T cells (58) as well as produce immunosuppressive cytokines (59). CAR-Tregs are independent of MHC and have high specificity, therefore they are promising candidates for therapy. Concurrently, CAR-Tregs can competitively bind IL-2 and inhibit the proliferation of effector T cells (60). Besides, CAR-Tregs are able to recognize the target antigen on target cells through the CAR structure and inhibit the function of effector T cells (61).

The pioneers of CAR-Treg research were Megan Levings and et al. The researchers developed a CAR specific to HLA-A2 and used it to generate Treg cells specific to human alloantigens (62). Experiments showed that Treg cells with an A2 CAR retain their phenotype and ability to suppress the immune response before, during, and after stimulation. In mouse studies, human Treg cells with an A2 CAR were shown to be more effective in preventing graft-versus-host disease than Treg cells with an irrelevant CAR.

CAR-Tregs have been used in the treatment of various disease models. CAR-Tregs with carcinoembryonic antigens (SCA431, scFv, CD28, and CD3z) have been used in the treatment of colitis and have shown their efficacy (63). Oligodendrocyte glycoproteins of myelin (MOG scFv, CD28, and CD3z) have been used in the treatment of multiple sclerosis demonstrating effective control of inflammatory responses (56). Besides, CAR-Tregs with insulin scFv, CD28, and CD3z molecules were used to treat a mouse model of T1DM and showed successful results without affecting the overall immune status of mice (64).

Treatments of AIDs using CAR-Tregs have not yet been tested in clinical trials. However, two registered clinical trials of CAR-Tregs have been reported, which were aimed to induce immune tolerance identical to the processes observed in AID. These trials were being conducted in another medicine area, namely in the transplantation of solid organs such as kidneys and livers, and involved CAR-Tregs targeting HLA-A2 (65, 66).

Concurrently, there are limitations in the use of CAR-Treg. In fact, CAR-Tregs are capable of migrating to the site of the inflammatory reaction to accomplish their suppressor functions by interacting with tissue-specific autoantigens (56, 67). If these autoantigens are expressed not only in the site of an autoimmune reaction but also in other healthy tissues of the body, the immune system response may be less effective and lead to systemic hyperactivation of CAR-Tregs that in turn can cause a non-specific immunosuppressive reaction (68).

A recently proposed approach to CAR-Treg optimization implies the production of universal CAR-Tregs (UniCAR-Tregs), which are designed on the basis of universal tumor-targeted CAR-T cells (UniCAR-T). UniCAR-T is a two-component system. The first component is a universal CAR-T cell that does not recognize human surface antigens but can interact with a peptide motif. This motif is contained in the second component, a soluble adapter called a targeting module (TM) (69). TMs are bispecific molecules that link UniCAR-T cells to target cells. UniCAR-T can be turned on and off by dosing the TMs (70). Unlike conventional CAR-T that target specific antigens on tumor cells, UniCARs are designed to recognize a non-immunogenic peptide epitope derived from the human La/SS-B protein. This design ensures that UniCAR-modified T cells remain inert until they encounter the appropriate target module that can bind to both the tumor cell and the UniCAR epitope (71). UniCAR-T have been used in clinical trials and have shown their effectiveness, for example in the study by Wermke et al. on the treatment of acute myeloid leukemia (69, 72). Regulation of the antigen specificity of UniCAR-Treg using a bispecific targeting module allows for an expansion of their scope of application. UniCAR-Tregs can be used universally, since their activation and antigen specificity are regulated by TM exchange (73). A study by Koristka et al. demonstrated that Tregs can be successfully modified and antigen-specifically activated using UniCAR technology both *in vitro* and *in vivo* (73).

Summarizing all the above, one can conclude that CAR-Tregs represent a promising approach to the treatment of AIDs, providing targeted immune modulation with potentially fewer side effects than traditional therapies. However, current research is essential to sort out the challenges associated with the implementation of this approach and ensuring its safe and effective use in clinical practice.

#### TCR-Tregs

In order to generate antigen-specific Tregs, researchers use retroviral or lentiviral transduction methodology to express antigen-specific TCR. These methods enable to obtain TCR-Tregs with the desired antigen specificity.

TCRs have been used to generate TCR-Tregs for the treatment of various AID models such as T1DM, multiple sclerosis, acquired factor VIII deficiency, etc. (74–77) and have shown their efficacy. In a study by Eggenhuizen et al., Sm-specific TCRs were transduced into Tregs, produced from SLE patients positive for anti-Sm and HLA-DR15. Sm-Tregs effectively suppressed inflammatory responses and inhibited disease progression in a humanized mouse model of lupus nephritis (78).

Antigen-specific Treg therapy has one distinct advantage over polyclonal Tregs. Tang et al. showed that a small amount of TCR-Tregs was sufficient to reduce disease activity and in some cases even completely reverse T1DM in NOD mice (79). Other researchers have also shown that only 2,000 TCR-Tregs were needed to prevent T1DM in mice (80). The lower dosage makes the antigen-specific TCR-Treg approach more advantageous in comparison to polyclonal Tregs.

Although relatively small numbers of antigen-specific Tregs may be required to ameliorate AID compared to polyclonal Tregs, identifying a suitable high-affinity antigen-specific TCR to transduce into Tregs remains a challenge in some AIDs. With a large diversity of TCRs, the number of Tregs in the peripheral blood is small. In addition, many Tregs reside in tissues, making their isolation challenging.

In clinical practice, the use of Tregs is associated with certain difficulties, since they need to be isolated from the total number of cells in the peripheral blood, where they make up approximately 1–5% of all CD4 lymphocytes (81). One approach to circumvent this obstacle is reprogramming T cells into Tregs. In particular, in the study by Wright et al., CD4^+^ T cells were transformed into antigen-specific Tregs using transduction of FOXP3 and a specific TCR targeted to serpin (82). This entailed the accumulation of TCR-transduced FOXP3 CD4^+^ T cells in draining lymph nodes, a decrease in the number of Th17, and the regression of bone tissue destruction (83). To obtain sufficient numbers of Tregs, researchers can also use the method of transforming antigen-specific effector T cells into induced Tregs through TGF-β and IL-2 stimulation, overexpression of transgenic FOXP3, blockade of cyclin-dependent kinase 8/19 (CDK8 and CDK19) signaling, and combination of overexpression of CTLA-4, IL-2 and antigen stimulation. For example, the combined use of a monoclonal antibody to CD3, a CDK8/19 inhibitor, and TGF-β resulted in a 10-fold increase in CD25^high^FoxP3^+^ Tregs in cultured CD4^+^ T lymphocytes compared to unstimulated lymphocytes due to the transdifferentiating of antigen-specific effector T lymphocytes into Tregs (84).

A key challenge in developing TCR-based therapies is to identify suitable target antigens that are expressed as peptides on specific HLA alleles (85). First, it is necessary to determine whether TCRs from Tregs or T effector cells are more promising sources for their isolation (86). Second, it may be necessary to identify TCRs restricted by widely expressed HLA molecules since not all AIDs are associated with HLA-DRB1 alleles, in particular. Third, sequence data from both TCRα and TCRβ will be needed to determine TCR specificity (87).

Another challenge in selecting TCRs is determining the required affinity. In this regard, low- and high-affinity TCRs have been compared in various studies (75, 88–90), which have shown that TCR affinity can be both positively (75) and negatively (89) correlated with immunosuppressive function. However, over time, it has become clear that there is a more complex pattern, in which the role of the TCR is context-dependent and may differ for both high- and low-affinity interactions. For example, researchers (90) showed that Tregs with high- and low-affinity TCRs migrated to the pancreas though Tregs with high-affinity TCRs activated classical pathways (CTLA-4, IL-10, etc.), while Tregs with low-affinity TCRs expressed proteins associated with tissue repair (amphiregulin and IL-35). It is worth noting that injection of both high- and low-affinity Tregs to NOD mice was more effective in suppressing disease activity than injection of only one of these types.

Studies on TCR-Tregs are associated with MHC class II. Presumably, TCRs of class I can be more effective since they are substantially distributed in tissues providing wider access to target cells. Some studies describe the use of high-affinity or low-affinity class I TCRs, specific for various tumor antigens (91–94). In particular, the study (72) emphasized the independence of TCR-Tregs on the CD8 coreceptor. Other authors (74) demonstrated that HLA-A2-restricted transduced Tregs, which did not express CD8, maintained FOXP3 expression, and suppressed tyrosinase-specific T cells. Another study (94) described CD4 coreceptor-independent Tregs, thereby explaining their ability to be stimulated by low-affinity class I TCRs without CD8, in contrast to CD4^+^ Tconv. All this increases the choice of cells for TCR selection enabling direct recognition of tissue antigens independently on APCs.

On the way from finding cells to isolate TCR to transduction and production of TCR-Tregs, researchers encounter another challenge related to improper coupling between the alpha and beta TCR chains of endogenous and transduced TCR. However, a solution to this problem is already being developed. In particular, to facilitate the correct coupling of TCR chains, it is common to use such methods as silencing of the endogenous TCR gene, CRISPR deletion of the endogenous TCR gene, modification of the transduced TCR by cysteinization of the alpha-beta constant chains through forming a disulfide bond (83, 95–98).

### Targeting inflammatory mediators

Recently, a group of scientists have presented a new concept for the production of Tregs with inflammation biosensors (99, 100). This approach may help to solve the problem of choosing antigens for Treg therapy. Inflammation biosensors are targeted at inflammatory mediators including TNFα, TNF-like ligand 1A, and tumor necrosis factor superfamily instead of antigens and are called artificial immune receptors (AIRs) (100). AIRs have an intracellular CD3z chain and a costimulatory signaling domain CD28 in their structure that may resemble the structure of CAR-T. AIRs have a number of advantages: activation of Tregs occurs only at the site of the inflammatory response and only membrane-bound ligands trigger the process; extracellular receptor domains interact with several ligands increasing efficacy; natural receptor domains are of high stability in comparison, for example, to CARs (100). Bittner et al. demonstrated that AIR-mediated activation of murine Tregs led to TCR-like signaling cascades and induction of Treg proliferation; moreover, AIR-Tregs were more efficient than polyclonal Tregs (100).

### Comparison of CAR-Tregs and TCR-Tregs

CAR-Tregs apply synthetic receptors designed to recognize specific antigens independently of MHC molecules (101), which enables their broader use across different patient groups without MHC compatibility limitations. CARs typically consist of a single-chain variable fragment (scFv) linked to intracellular signaling domains, which activate Tregs upon antigen binding (100, 102). However, if there is a lack of surface molecules, CAR may not work. Besides, the ability to target intracellular antigens is blocked due to MHC-independent antigen recognition (103, 104). On the other hand, TCR-Tregs rely on T cell receptors, which require specific MHC-antigen complexes for activation. This may create limitations in efficacy among different patient populations, as the TCR must match the patient’s MHC type (55, 102).

CAR-Tregs have a high affinity for their cognate antigen but for recognition and activation of Tregs, a large number of target antigens on the surface of target cells are required (105). In contrast, TCR-Tregs require only one interaction of the peptide–MHC with the TCR to activate Tregs (106–109).

CAR-Tregs have been shown to exhibit increased cytotoxicity and a more inflammatory profile compared to TCR Tregs. They secrete higher levels of inflammatory cytokines, which may result in declined suppression of effector T cells and APCs (55, 110). These processes may enhance their ability to target specific antigens but carry the risk of unwanted inflammatory reactions. In contrast, TCR-Tregs maintain a more stable immunosuppressive function due to their dependence on natural signaling pathways associated with endogenous activation of TCR. They tend to have a less inflammatory phenotype and are more effective in suppressing immune responses without causing excessive activation of T effector cells (102, 111).

Concurrently, there are general disadvantages of using these cell immunotherapy strategies, which include high production costs and problematic target selection. It is also necessary to take into account that the main side effects will only be determined with the widespread use of these approaches. One of the possible side effects upon injecting these cells may be the loss of FOXP3 expression in inflammatory foci of the environment, in which induced Tregs can be transformed into effector T cells, promoting the aggravation of the disease. Several approaches are known to stabilize the Treg phenotype including treatment of Tregs with all-trans retinoic acid (ATRA) and stimulation of ectopic expression of the FOXP3 gene to stabilize the regulatory phenotype (112).

## Treg exosomes

Exosomes are extracellular vesicles, which transport bioactive molecules for intercellular communication (113). Exosomes can transfer proteins, lipids, and nucleic acids (DNA, mRNA/miRNA) to target cells (114). They are widely distributed in body fluids and are involved in intercellular communication, homeostasis, and angiogenesis (115, 116). The integral structure of exosomes enables to keep their molecular cargo intact when crossing biological barriers; that is crucial for targeting specific tissues (117, 118). Exosomes are highly stable under various storage conditions, which simplifies their handling and increases shelf life compared to traditional cell therapy. They can be lyophilized to produce ready-made preparations, which facilitates their use in clinical settings without the need for immediate administration (119). Thus, exosomes are promising delivery agents for drugs and bioactive molecules.

The immunosuppressive function of Treg exosomes may be mediated by their CD73 content, which promotes adenosine production (120). It is currently unknown whether Treg exosomes contain immunoregulatory cytokines IL-10, IL-35, and TGF-β. However, there is other interesting evidence: exosomes derived from FOXP3^+^ Treg cells contain various microRNAs. In a study by Okoye et al., microRNAs and the exosomal pathway were shown to be essential for effective Treg cell function (121). Let-7d was found to be packaged and transported to Th1 cells, suppressing Th1 cell proliferation and IFN-γ secretion.

Yu et al. investigated the use of Treg exosomes as a therapy in transplantation (122). The authors isolated and purified Treg-derived exosomes and established a rat kidney transplant model. Then, autologous exosomes were administered to recipients. The study showed that exosome function could delay allograft rejection and prolong the survival time of the transplanted kidney by suppressing T cell proliferation.

Some studies confirm that exosomes isolated from Tregs have functions similar to Tregs, namely they are able to suppress autoimmune reactions and induce immunological tolerance (123, 124).

Thus, exosomes represent a compelling alternative to Tregs due to their ability to safely and effectively modulate immune responses, versatility in therapeutic applications, and practical advantages in production and storage. However, there is a downside: the potential side effects and mechanisms of action of Treg exosomes are not fully understood. As research progresses, exosome-based therapies can revolutionize treatment strategies in various areas of medicine.

## Conclusion

Both polyclonal and antigen-specific Tregs are used for the treatment of AIDs. Many studies confirm that antigen-specific Tregs are more effective even upon using a small dosage of the cells in comparison to polyclonal Tregs. Currently, the approaches with CAR-Tregs and TCR-Tregs are being actively developed. Both types of cell therapy are quite promising, but it is still necessary to solve many problems in designing and determining sources for cell production, dosages and methods of administration for further use in clinical trials.

## Future prospects

The field of Treg therapy is rapidly evolving, especially with the emergence of engineered Tregs such as CAR-Tregs and TCR-Tregs. These methods will enable the treatment of various AIDs, transplant rejection, and a number of other immune-mediated disorders. Promising data have been obtained upon the use of CAR-Tregs and TCR-Tregs in preclinical disease models. However, before moving on to the clinical stage, many experiments will need to be conducted to improve different stages of cell production, including methods of their obtaining and stabilization. Perhaps a new direction of Treg therapy will be a combination of different methods, for example, the combined use of TCR-Tregs and rapamycin or low doses of IL-2. Besides, combinations of CAR-Tregs or TCR-Tregs with exosomes derived from Tregs are also possible, as well as the combined use of CAR-Tregs and TCR-Tregs for eliminating drawbacks of each of them and affecting different targets. Moreover, a promising direction may be associated with understanding the specificity of the type of cell therapy for an individual patient.

Finding a suitable source for TCR remains a common problem. A successful strategy can be generating antigen-specific CD8^+^ T cells followed by the isolation of TCR from them for subsequent viral transduction into T cells together with FOXP3. In addition, a future-oriented direction may be the generation of antigen-specific Tregs for TCR isolation using DCs modified with DNA constructs, as has been shown in our previous work (125).

The future of TCR-Treg and CAR-Treg approaches looks promising, even with current advances in research aimed at overcoming limitations and improving therapeutic efficacy.
